# Impact of Obesity on Quality of Life and Owner’s Perception of Weight Loss Programs in Cats

**DOI:** 10.3390/vetsci8020032

**Published:** 2021-02-20

**Authors:** Rachel Hanford, Deborah E. Linder

**Affiliations:** Cummings School of Veterinary Medicine, Tufts University, North Grafton, MA 01536, USA; rachel.hanford@tufts.edu

**Keywords:** obesity, cats, quality of life, nutrition

## Abstract

Obese dogs have been shown to have a diminished quality of life; however, there is less evidence characterizing the impact of obesity on the quality of life of cats. A cross-sectional survey study was conducted among cat owners with either healthy weight cats (body condition scores of 4–5/9) or obese cats (body condition scores of 8–9/9) as determined by a veterinarian. Exclusion criteria included medical conditions (determined by physical exam and screening bloodwork). Cat owners completed surveys on quality of life and perceptions of feline obesity. Quality of life scores for obese cats had a wider range and were numerically lower compared to scores of healthy weight cats with a moderate effect size of 0.68, though this was not a statistically significant difference (71.2 ± 8.8 vs. 75.9 ± 4.1, *p* = 0.0881; n = 33). Owners of obese cats less frequently reported that obesity was a high risk to their cat’s health (77% [10/13]) vs. 100% [20/20]) and less frequently cited the primary caretaker as a cause of cat obesity (30% [3/10] vs. 55% [11/20]) compared to the owners of healthy weight cats. Interestingly, 97% (32/33) of all owners believed veterinarians should play a role in feline weight loss. These results suggest that some obese cats can have a potentially diminished quality of life but highlights the need for more data surrounding the impact of feline obesity and enhanced client communication strategies to best address obesity in the feline population.

## 1. Introduction

Obesity, defined as a body condition score (BCS) of 8–9/9 [1], is a widespread and increasing problem for companion animal populations with potentially devastating health complications. Although many physical health consequences of obesity have been researched in companion cat and dog populations, there is less understanding of how obesity impacts quality of life, and how quality of life should be defined and assessed, in the up to 60% of cats in the United States affected [2,3,4,5]. Previous studies have investigated the relationship between feline body condition score and obesity-related comorbidities [6], as well as owner-perceived happiness [7]. Additionally, a study in dogs found obesity negatively impacted quality of life, though improved upon weight loss [8]. However, there is a lack of evidence on the relationship between obesity and owner-perceived quality of life in cats with no other medical conditions.

Further understanding the impact of obesity among cats would allow veterinarians to better communicate the urgency for proper nutrition and body condition to pet owners, particularly if the quality of life was negatively impacted. Thus, to better characterize the impact of obesity in the feline population and provide enhanced opportunities for client communication, the objectives of this study were to determine the impact of obesity on quality of life in companion cats and to examine owner perceptions of feline obesity. 

## 2. Materials and Methods 

### 2.1. Study Design and Population Criteria

A sample size calculation determined that 30 participating cats would be necessary to achieve a power of 80% and a level of significance of 5% (two-sided) to detect a 15% difference in quality of life scores between ideal weight and obese cats based on the expected difference seen in validity studies of this questionnaire in cats with owner-reported “good–fair” and “excellent” health [9]. A total of 36 animals were included in the study to maintain adequate sample size while allowing for some attrition due to the detection of comorbidities during the eligibility screening.

Owners of adult cats with healthy weights (BCS: 4–5/9) or obese (BCS: 8–9/9) were eligible to participate in the study. BCS of both 4/9 and 5/9 were categorized as healthy weight based on the original body condition validation determining 5/9 as ideal [10] and more recent research highlighting the need for ideal BCS, especially for neutered cats, to include a BCS of 4 [11]. Exclusion criteria were cats less than one year of age, BCS of 1–3 and 6–7, or medical conditions reported by owners or diagnosed through screening bloodwork or physical exam (apart from excessive body weight).

### 2.2. Data Collection

The study was conducted at Henry and Lois Foster Hospital for Small Animals in central Massachusetts. Participants were recruited with a “snowball sampling” technique through an email sent to local veterinarians as well as all Tufts employees and students on the Grafton university campus. Recruitment also occurred through online digital flyers (e.g., posts on social media) advertising the study and a USD 25 gift card incentive. 

Participants were asked to self-report their cat’s quality of life as measured by a previously validated 16-item scale for feline overall health-related quality of life [9]. Alternative quality of life measurements (33 items and eight domains) are available [12], though the questionnaire in this proposed study (16 items) was chosen due to its potential as a rapid assessment for general practitioners to use in the future. Questions included a scaled reporting of the frequency of healthy behaviors, such as purring, vocalizations, and grooming, as well as clinical health indicators (e.g., illness, energy-levels, weight-loss, pain) over a 4-week period (e.g., “In the past four weeks, my cat has…”). A few example questions were “In the past four weeks, my cat has been curious and shown an interest in its surroundings (e.g., looking outdoors, watching surroundings, looking under furniture, sniffing objects)” and “In the past four weeks, my cat has made sounds indicating happiness (e.g., purring, meowing, ‘talking’).” Owners could select between five answers for each behavior frequency including “Not at all,” “A little,” “Somewhat,” “Quite a bit,” or “A great deal.” Owners were also provided a series of exploratory questions to better understand personal motivators for pet weight loss and preferred interventions (see Supplemental Materials). This included identifying the perceived cause of feline obesity, qualifying obesity as a potential health risk, and the perception of weight loss interventions that would be appropriate for their pet. A demographic questionnaire was also included to provide information on the sex, race, household income, and education level of the participants. 

A physical exam with BCS [10], muscle condition score, and body weight assessment was conducted by a Board Certified Veterinary Nutritionist^®^, and fasting bloodwork was performed which included a complete blood count and chemistry profile analysis. This screening was used to confirm eligibility and identify possible comorbidities independent of excessive body weight that could impact quality of life.

### 2.3. Statistical Analyses

Data were graphically assessed for normality and are presented as the mean ± standard deviation for normally distributed data and median (range) for skewed data (Table 1 and Table 2). Analyses were performed within SPSS. A *p*-Value <0.05 was considered statistically significant. The quality of life score was analyzed using a two-sample *t*-test. Demographic differences between groups were measured using Wilcoxon signed rank tests for continuous data (e.g., age, weight, etc.) and chi-square tests or Fisher’s exact tests (if expected frequencies of <5) for categorical data (e.g., household income, race, etc.). Qualitative questionnaire answers were evaluated using descriptive statistics.

## 3. Results

### 3.1. Population Data

Of the 43 cats screened, seven were excluded due to a BCS of 6/9 or 7/9. Three cats were excluded due to the presence of comorbidities (aside from excessive body weight) found on physical exam, complete blood count, and/or blood chemistry panel. Of the remaining 33 cats, 39.4% (n = 13) were categorized as obese (BCS 8–9/9) and 60.6% (n = 20) were categorized as healthy weight (BCS 4–5/9), Table 1. 

There were no significant differences in owner demographics between the two groups with the exception of race; as the percentage of cat owners who identified as non-White was higher among cat owners with healthy weight cats compared to cat owners with obese cats (*p* = 0.039); however, only five non-White owners participated in the study. Cat characteristics were similar between the two groups aside from higher weight and higher BCS in the obese group (*p* < 0.001), Table 1. All cats were neutered, and physical exam and laboratory work revealed no significant abnormalities on any of the 33 cats included in the study (with the exception of obese BCS).

### 3.2. Quality of Life and Qualitative Question Data

Quality of life scores for obese cats had a wider range and were numerically lower compared to the scores of healthy weight cats with a moderate effect size of 0.68, though this was not a statistically significant difference (71.2 ± 8.8 vs. 75.9 ± 4.1, *p* = 0.0881; n = 33), Table 2.

Owners of obese cats less frequently reported that obesity was a high risk to their cat’s health (77% [10/13]) vs. 100% [20/20]) and less frequently cited the primary caretaker as a cause of cat obesity (30% [3/10] vs. 55% [11/20]) compared to owners of healthy weight cats. A large majority, 85% (11/13), of owners of obese cats said they would implement a weight loss plan for their cat, but only 47% (6/13) had done so previously. Furthermore, two (out of 13) owners of obese cats stated they would not initiate a weight loss plan for their cat at all compared to 1 (out of 20) owners of healthy weight cats.

Of the 30 owners (11 obese cats and 19 healthy weight cats) that would consider a weight loss program for their cat, 53% (16/30) would do so if their cat gained a significant amount of weight, with the remaining participants only choosing to initiate a program on the advice of a veterinarian (33% [10/30]), if their cat developed a medical problem (3 out of 30), or a combination of the above (1 out of 30). Nearly all of these owners that would pursue a weight loss program cited health and quality of life concerns as motivations (97% [29/30]) and expressed an intervention preference for diet and exercise plans (93% [28/30]) over medications/supplements (3 out of 30), support groups (2 out of 30), and surgical interventions (3 out of 30). In addition, 97% (32/33) of all participants believed that veterinarians should play a role in weight loss for cats.

## 4. Discussion

Though the main findings of the study do not show a statistically significant difference in owner-perceived quality of life scores between obese and healthy weight cats, the findings do highlight a larger range of and a numerically lower mean quality of life score among obese cats compared to healthy weight cats. These findings suggest that some obese cats can have a potentially diminished quality of life and given the particularly larger variance of quality of life scores among obese cats, may also suggest that the impact of obesity on quality of life is more variable than has previously been shown in dogs, ref. [8] with obesity impacting some cats differently than others. In addition, the qualitative assessment performed helps to increase understanding of owner perceptions and motivations related to obesity and weight loss interventions cats. 

Quality of life is a complex assessment of behavior and health factors, of which obesity may serve as only a partial contributor. This is best supported by the large variance in quality of life scores in the obese group. Additionally, these complications may also contribute to why even though 85% (11/13) of owners of obese cats said they would implement a weight loss plan for their pet, only 47% (6/13) had attempted to do so prior to taking the survey. As such, it is possible that important indicators of companion cat quality of life may have been overlooked. Alternative and more comprehensive quality of life measurements were available [12], though the questionnaire used in this proposed study was chosen due to its potential as a rapid assessment that general practitioners might be able to use in private practice to improve client understanding of the effects of obesity on cats.

The present study also highlights a concerning gap in communication between veterinarians and clients on the topic of feline nutrition, weight management, and pet obesity. Though 85% of owners of obese cats in this study reported they would implement a weight loss plan for their cat, only 47% had done so previously, emphasizing a gap between owner intentions and actions that the veterinary healthcare team could address. Promisingly, 97% of participants in the study believed that veterinarians should play a role in weight loss for cats. Since almost every owner (97%) in this study that would pursue a weight loss program cited health and quality of life concerns as motivations for doing so, this study underlines the need for veterinarians to discuss the potential impact of obesity on health and quality of life with cat owners. Given the wide range of quality of life scores seen among obese cats in this study, veterinarians could provide quality of life questionnaires for cat owners as a communication tool to better align veterinary companion cat health goals with that of pet owners. Interventions could also be introduced to pet owners and tailored to the expressed preferences shown in this study such as including their veterinarian (97%), focusing on diet and exercise (93%), and avoiding medications, supplements, support groups, and surgical interventions.

Limitations of the study must be considered, such as its generalizability and sample size, since this population only reflects those pet owners whose geographic proximity to the Henry and Lois Foster Hospital for Small Animals allowed them to participate in the required study appointment. It is also important to note that many participants were students or staff at Tufts University. The smaller sample size may limit the evaluation of the survey method for analyzing obesity-related changes in quality of life and may not be generalizable to all obese cats. A sample size calculation originally determined that 30 participating cats would be necessary to detect a statistically significant difference in quality of life scores between ideal weight and obese cats based on the expected difference seen in validity studies of this questionnaire in cats [9]. However, the range of scores seen in this study were much more variable than that seen in the previous validation study, particularly among the obese cats. An effect size calculation was performed to better interpret these results and revealed a moderate effect size of 0.68, suggesting that this unexpected variance led to an inaccurate sample size calculation and the lack of significance could be due to a smaller sample size. Future studies could utilize this new data to more accurately design a future study to confirm these potential findings. 

In addition, pet owner-based assessments may be biased due to individual differences in interpretation even with the use of a previously validated method and this could be mitigated with more objective methods of evaluation including assessment by a veterinarian. Further behavioral research on cats may also serve to better identify important factors in objectively evaluating feline quality of life.

Future studies could also expand the study population to a more diverse racial/ethnic background and geographic population of participants to further understand a variety of owner perceptions and motivations surrounding feline obesity and weight-loss interventions. In addition, cat characteristics (age, sex, etc.) and covariates that could affect perceptions of quality of life, such as the duration of ownership, or number of co-habiting cats, could be addressed as well as the veterinarian’s assessment of quality of life including nutritional management and degree of sedentary lifestyle. 

The current study demonstrates a numerical decrease and a larger variance in quality of life scores among obese cats, highlighting the potential for obesity to negatively impact quality of life in cats. Owner perceptions documented in the qualitative questionnaire such as intention but not action to initiate weight loss plans also highlight the need for client communication about nutrition, weight loss interventions, and obesity. Future studies are warranted to evaluate the impact of including quality of life concerns in cat dietary interventions, as well as to determine if quality of life improves following weight loss. Overall, the study highlights the need for more data surrounding the impact of feline obesity and enhanced client communication strategies to best address obesity in the feline population. 

## Figures and Tables

**Table 1:** Demographic information of the human and feline study population expressed in median (range) or count (percentage) between obese and healthy weight groups.

Variable	Obese	Healthy Weight	*p* Value
n	13	20	—
Pet Owner Characteristics			
Age (year)	41.5 (24–69)	32.7 (22–59)	0.058
Sex			0.772
Female, n (%)	12 (92.3%)	19 (95.0%)	
Education Level			0.772
High School or Less	1 (7.7%)	1 (5.0%)	
Household Yearly Income Level			0.298
Less than USD 19,999, n (%)	0 (0.0%)	6 (30.0%)	
USD 20,000–39,999, n (%)	2 (15.4%)	3 (15.0%)	
USD 40,000–59,999, n (%)	5 (38.5%)	1 (5.0%)	
USD 60,000–79,999, n (%)	0 (0.0%)	1 (5.0%)	
USD 80,000–99,999, n (%)	0 (0.0%)	2 (10.0%)	
USD 100,000+, n (%)	4 (30.8%)	6 (30.0%)	
Race			0.039
White	13 (100%)	15 (75.0%)	
Hispanic or Latino	—	1 (5.0%)	
Black or African American	—	1 (5.0%)	
Asian/Pacific Islander	—	2 (10.0%)	
Other	—	1 (5.0%)	
Cat Characteristics			
Age (y)	7.08 (1–15)	5.31 (1.5–12)	0.210
Weight (kg)	7.26 (4.7–8.6)	4.46 (2.7–6.6)	<0.001
Body Condition Score	8.69 (8–9)	5.0 (4–5)	<0.001

**Table 2:** Comparison of quality of life scores between obese and healthy weight cats.

Variable (Mean)	Obese	Healthy Weight	*p* Value
n	13	20	—
Quality of Life Score	71.2 ± 8.8	75.9 ± 4.1	0.088

## Data Availability

The data presented in this study are available on request from the corresponding author.

## Supplementary Materials

The following are available online at https://www.mdpi.com/2306-7381/8/2/32/s1, Owner-Perceived Impact of Obesity on Quality of Life in Cats.
