# Supplementary material for: Impact of Obesity on Quality of Life and Owner’s Perception of Weight Loss Programs in Cats
Source: Vet Sci. 2021 Feb 20;8(2):32. doi: 10.3390/vetsci8020032 (PMC7924056; doi:10.3390/vetsci8020032)
Supplement: Supplementary file 1 [file vetsci-08-00032-s001.pdf]

Owner-Perceived Impact of Obesity on Quality of Life in Cats
[Logic was used so only applicable persons were shown the question. Questions that used logic are marked with an asterisk]

Cat information

Q2.1 Please enter information on the cat brought to this appointment:
Q2.2 Breed: ____

Q2.3 Age: ____

Q2.4 Sex:
- o Male
- o Female

Q2.5 Neutered:
- o Yes
- o No

Q2.6 Do you consider your cat:
- o Underweight
- o Ideal Weight
- o Overweight

Q2.7 Have you ever tried to get your cat to lose weight?
- o Yes
- o No

Q2.8 If yes, what strategies did you use:*
- o Changing to a diet food I could get over-the-counter (grocery or pet store).
- o Changing to a prescription diet food I could only get from my vet (or online).
- o Changing or reducing treats.
- o Increasing exercise/physical activity.
- o Working with a vet or vet technician to develop a weight loss program.
- o Giving a weight loss medication.
- o Other/details: ____

*only shown to participants that selected yes to question 2.7

Owner opinions on obesity in cats

Q3.1 What percentage of cats do you think are overweight? (___%)
____

Q3.2 In your opinion, what is the primary cause of cat obesity (please select best answer)?
- o Primary caretaker/myself.
- o All of the family/others in the household.
- o High calorie cat food and treats.
- o Cat's appetite or hunger.
- o Other/details:____

Q3.3 What risk do you think being overweight has on a cat's health (please select best answer)?
- o None or slight risk
- o Moderate Risk
- o High Risk
- o Extremely High Risk

Q3.4 Do you believe veterinarians have a role in cat weight loss?
- o Yes
- o No

Q3.5 If yes, what is that role (check all that apply)?*
- o Telling me when my cat is overweight.
- o Working with me to create a comprehensive diet and exercise plan.
- o Providing me with diet food recommendations.
- o Helping me with any problems I have trying to get my cat to lose weight.
- o Other/details:____

*only shown to participants that selected yes to question 3.4

Q3.6 The rest of the questions refer specifically to your cat. Please consider your own cat (the one you noted above) when answering the following questions.

Q3.7 Would you ever institute a weight loss plan for your cat if your cat was overweight?
- o Yes
- o No

Q3.8 When would you start a weight loss plan for your cat (please select best answer)?*
- o If my cat gained a lot of weight.
- o If my veterinarian told me I should.
- o If my cat had a medical problem (diabetes, arthritis, etc.).
- o Other/details:____

*only shown to participants that selected yes to question 3.7

Q3.9 What would be your motivation to start a weight loss plan for your cat (please select best answer)?*
- o Health concerns.
- o Appearance/embarrassment concerns.
- o Physical limitation or fitness concerns.
- o Quality of life concerns.
- o Other/details:____

*only shown to participants that selected yes to question 3.7

Q3.10 Which would you consider as part of a weight loss plan for your cat (check all that apply)?*

- Changing to a diet food I could get over-the-counter (grocery or pet store).
- Changing to a diet food I could only get from my vet (or online).
- Changing or reducing treats.
- Increasing exercise/physical activity.
- Working with a vet or vet technician to develop a weight loss program.
- Giving a weight loss medication.
- Having my cat undergo weight loss surgery (e.g., gastric bypass or gastric band).
- Herbal or natural supplement.
- Other/details:____

*only shown to participants that selected yes to question 3.7

Q3.11 Which of these strategies would be most effective for your cat (please select best answer)?*

- Changing to a diet food I could get over-the-counter (grocery or pet store).
- Changing to a diet food I could only get from my vet (or online).
- Changing or reducing treats.
- Increasing exercise/physical activity.
- Working with a vet or vet technician to develop a weight loss program.
- Giving a weight loss medication.
- Having my cat undergo weight loss surgery (e.g., gastric bypass or gastric band).
- Other/details: ____

*only shown to participants that selected yes to question 3.7

Q3.12 What services or tools would be helpful for you if you wanted to address your cat's weight (check all that apply)?*

- Low calorie food or treats that my cat liked.
- A support group.
- An exercise program.
- Supplements to make my cat less hungry or beg less.
- Medications to make my cat less hungry or beg less.
- Other/details: ____

*only shown to participants that selected yes to question 3.7

Participant information

Q5.1 Please answer these questions about yourself.
Q5.2 Gender
- o Male
- o Female

Q5.3 What is your age? _____ yrs

____

Q5.4 What is the highest level of education you have completed?
- o Some high school
- o 12th Grade or GED
- o Some college/Associate Degree
- o College
- o Non-doctoral graduate degree
- o Doctoral Degree (MD, PhD, etc.)

Q5.5 What is your total income of your household?
- o Less than $19,999
- o $20,000-$39,999
- o $40,000-$59,999
- o $60,000-$79,999
- o $80,000-$99,999
- o $100,000 and above
- o Prefer not to answer

Q5.6 What is your zip code?

____

Q5.7 Please specify your ethnicity:
- o White
- o Hispanic or Latino
- o Black or African American
- o Native American or American Indian
- o Asian / Pacific Islander
- o Other____
- o Prefer not to answer

Q5.8 Thank you again for your time. We appreciate your assistance in helping us learn how to best treat pets. If you have any further comments or feedback, feel free to add them in the space below.
